# The complete mitochondrial genome of *Apis mellifera meda* (Insecta: Hymenoptera: Apidae)

**DOI:** 10.1080/23802359.2017.1325342

**Published:** 2017-05-12

**Authors:** Amin Eimanifar, Rebecca T. Kimball, Edward L. Braun, Stefan Fuchs, Bernd Grünewald, James D. Ellis

**Affiliations:** a Honey Bee Research and Extension Laboratory, Entomology and Nematology Department, University of Florida, Gainesville, FL, USA;; b Department of Biology, University of Florida, Gainesville, FL, USA;; c Institut für Bienenkunde, Polytechnische Gesellschaft, Goethe-Universität Frankfurt am Main, FB Biowissenschaften, Oberursel, Germany

**Keywords:** Mitogenome, next-generation sequencing, *Apis mellifera meda*

## Abstract

The complete mitochondrial genome of the western honey bee subspecies *Apis mellifera meda* was sequenced. This mitochondrial genome is 16,248 bp in length, with 37 classical eukaryotic mitochondrial genes and an A + T-rich region. Gene direction and arrangement are similar to those of other *Apis* mitogenomes. All genes initiate with ATT (six genes), ATG (four genes), ATA (two genes), and ATC (one gene) start codons and terminate with a TAA stop codon. Four genes are encoded on the heavy and nine on the light strands, respectively. All of the 22 tRNA genes, ranging from 66 to 78 bp, have a typical cloverleaf structure. The complete mitogenome of *A.m. meda* provides information on the biogeography and evolution of *A. mellifera* subspecies.

*Apis mellifera meda* is a subspecies of western honey bee native to Iran, northern Iraq, and southeastern Turkey (Sheppard 2015). *Apis mellifera meda* shares similarities with *A.m. ligustica*, *A.m. anatolica*, and honey bees in northern Iraq (Rahimi & Asadi 2010) and is similar, morphometrically, to *A.m. ligustica*, the Italian honey bee (Sheppard 2015). The complete mitochondrial genome of *A.m. meda* has not been studied. In this study, an adult honey bee worker of *A.m. meda* was obtained from the Ruttner Bee Collection at the Bee Research Institute at Oberursel, Germany (Voucher No. 3284, Pour-Elmi, Iran, 2004, 27°13N, 60°40E) and its mitogenome is reported (GenBank accession No. KY464957). The identity of the bee was confirmed morphometrically by the Institute staff. We followed the method of Eimanifar et al. (2016) to extract and quantify genomic DNA. A genomic library was constructed from the genomic DNA using a Kapa Hyper Prep Kit (Kapa Biosystems, Woburn, MA) with a paired-end read (2 × 150) followed by next-generation sequencing on the Illumina Hi-Seq 3000/4000 (San Diego, CA). The mapping, assembly and annotation were performed as described previously (Eimanifar et al. 2017).

The complete sequence of *A.m. meda* is 16,248 bp in length and consists of 13 protein-coding genes (PCGs), 22 transfer RNA (tRNA) genes, 2 ribosomal RNA (rRNA) genes, and 1 putative control region (CR). The overall base composition of the *A.m. meda* mitogenome was 43.1% A, 41.7% T, 9.6% C, and 5.6% G. The gene content, structure, and arrangement of the *A.m. meda* mitogenome are similar to those observed in other *Apis* mitogenomes (Eimanifar et al. 2016), with four PCGs on the heavy strand with the remainder on the light strand. ATP6 and ATP8 share 19 nucleotides, though other PCGs do not overlap. Start codons included ATT (six PCGs), ATG (four PCGs), ATA (two PCGs), and ATC (one PCG), with all ending in a TAA stop codon.

The 16S rRNA and 12S rRNA were 1362 and 785 bp long, respectively, with 16S having a slightly higher AT content (84.4% versus 81.5%). These are located between the tRNA-Leu gene and CR, and were separated by the tRNA-Val gene. The 22 tRNAs all fold into typical a cloverleaf shape as identified by tRNAscan-SE (Lowe & Eddy 1997), and ranged in length from 66 to 78 bp. The 763 bp CR was 96.2% AT.

The phylogenetic position of *A.m. meda* was estimated from a concatenated dataset including the 13 PCGs and 2 rRNAs using the software RAxML 8.2.0 on the CIPRES science gateway (Miller et al. 2010) with GTRGAMMA and 1000 bootstrap replicates (Figure 1). In the future, it is necessary to sequence the mitochondrial genomes of other *A. mellifera* subspecies to determine the phylogenetic relationships between them (especially the Middle Eastern subspecies) and *A.m. meda.* The *p*-distance between *A.m. meda* and *A.m. liguistica* is 0.004, while it ranged from 0.013 to 0.014 with *A.m. mellifera* individual. Additional mitogenomes from unstudied *Apis* subspecies will promote our understanding of the mitogenome evolution and diversity in *Apis*.

**Figure 1.**
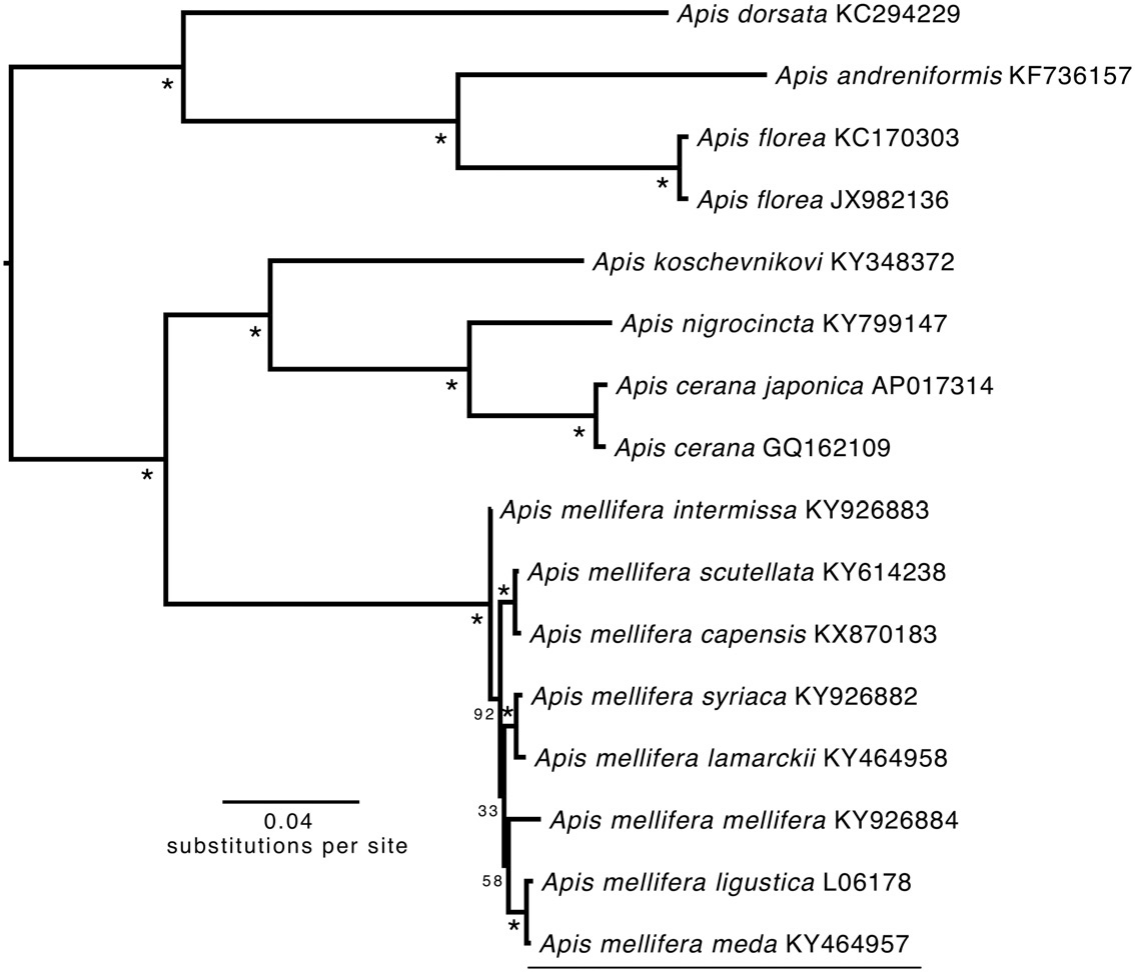
Phylogenetic tree showing the relationship between *A.m. meda* and 15 other *Apis* species and subspecies based on maximum-likelihood (ML) approach. Numbers behind each node denote the bootstrap support values. All 15 GenBank accession numbers are mentioned after the scientific names.
